# Neutrophil Count, Intracranial Atherosclerotic Stenosis, and Prognosis of Ischemic Stroke After Endovascular Treatment: A Mediation Analysis

**DOI:** 10.3389/fneur.2020.605852

**Published:** 2020-12-18

**Authors:** Tingting Li, Zhonglun Chen, Xuyin Zhu, Xianbiao Tang, Song Pan, Fan Gong, Leyi Xu, Mingzhe Wang, Hongzhi Zhang, Yongmei Guo, Jingsi Zhang, Baofeng Qin, Zongqi Zhang, Yun Liu, Zhimin Fei, Weidong Pan, Xiaofei Yu, Dezhi Liu

**Affiliations:** ^1^Department of Neurology, Shuguang Hospital Affiliated to Shanghai University of Traditional Chinese Medicine, Shanghai, China; ^2^Institute of Neurology, Shuguang Hospital Affiliated to Shanghai University of Traditional Chinese Medicine, Shanghai, China; ^3^Department of Neurology, MianYang Central Hospital, Mianyang, China; ^4^Department of Rehabilitation Medicine, Puer Hospital of Traditional Chinese Medicine, Pu'er, China; ^5^Medical Imaging Department of Shanxi Medical University, Taiyuan, China; ^6^Department of Neurosurgery, Shuguang Hospital Affiliated to Shanghai University of Traditional Chinese Medicine, Shanghai, China

**Keywords:** stroke, endovascular treatment, neutrophil, intracranial atherosclerosis, mediation analysis

## Abstract

**Background and Purpose:** Data on the relationship among neutrophil count, intracranial atherosclerotic stenosis (ICAS), and functional outcomes after endovascular thrombectomy (EVT) for ischemic stroke patients remains unclear. We aimed to evaluate the association between neutrophil count and prognosis of EVT patients and to determine whether the association was mediated by ICAS.

**Methods:** We retrospectively analyzed consecutive patients who underwent EVT at two comprehensive stroke centers between June 2016 and December 2019. A remaining stenosis >70%, or a lesser degree of stenosis with a tendency toward re-occlusion or flow impairment during the procedure, was classified as ICAS. A poor outcome was defined as a 90-day modified Rankin Scale score of 3–6.

**Results:** Of the 221 patients (mean age, 65.9 years; males, 61.1%) included in this study, 81 (36.3%) had ICAS, and 120 (54.3%) experienced a poor outcome at 90 days, respectively. In the multivariate adjustment for potential confounders, neutrophil count (odds ratio [OR], 1.19; 95% confidence interval [CI], 1.04–1.36; *P* = 0.012) and presence of ICAS (OR, 2.65; 95CI%, 1.28–5.45; *P* = 0.008) were risk factors of poor outcomes. Furthermore, mediation analysis indicated that total ICAS mediated the association between increased neutrophil count and worse functional outcome after EVT (the regression coefficient was changed by 11.7% for poor outcome, and 17.1% for modified Rankin Scale score, respectively).

**Conclusions:** Our study demonstrated that a higher neutrophil count might increase the risk of a poor outcome among ischemic stroke patients who underwent EVT, which was partially mediated by ICAS.

## Introduction

Several randomized controlled trials and meta-analyses have confirmed that endovascular thrombectomy (EVT) with stent retriever was a safe and effective way or achieving reperfusion in anterior circulation ischemic stroke caused by occlusion of the proximal anterior artery (1–5). Large-artery occlusion is generally due to embolism from proximal sources in Caucasians, whereas intracranial atherosclerotic stenosis (ICAS) with *in situ* thrombus occlusion are much more common in Asian patients (6). ICAS may challenge the passage of the retriever devices to the targeting lesions (7). Moreover, EVT with stent retrievers for ICAS may produce blood vessel injury and lead to re-occlusion caused by subsequent platelet aggregation at the site of the intracranial atherosclerotic lesion (8). Therefore, uncertainties remain regarding the benefit of EVT in patient with ICAS-related occlusion, especially in the Chinese population.

Neutrophils, as the indicator of acute and chronic inflammation, are the first cells that migrate from the peripheral vessel into the brain ischemic zone after stroke (9). The increased neutrophil count may accelerate inflammatory response by releasing proinflammatory cytokines and chemokines, in which the ischemic injury is intricately aggravated (10). Recent experimental studies have also found that acute ischemic stroke thrombi contain neutrophil extracellular traps and that their numbers were associated with intravenous thrombolysis and EVT resistance (11, 12). Neutrophils are not only associated with clinical outcomes in ischemic stroke after EVT (13), but also play a pivotal role in ICAS (14). Even in a healthy population, the baseline neutrophil-lymphocyte ratio was reported to be linked to the progression of ICAS (15). However, the relationship between neutrophil count, presence of ICAS, and functional outcomes after EVT is still unclear. We therefore performed this retrospective multicenter cohort study to assess the association between neutrophil count and prognosis of ischemic stroke patients treated with EVT, and to determine whether the association was mediated by ICAS-related occlusion.

## Methods

### Study Design and Subjects

We retrospectively analyzed consecutive ischemic stroke patients who underwent EVT at two comprehensive stroke centers (Shuguang Hospital and Mianyang Central Hospital) between June 2016 and December 2019. Patients were recruited in this study if they: (1) had acute intracranial large artery occlusion of the anterior circulation confirmed by computed tomographic angiography, magnetic resonance angiography, or digital subtracted angiography (DSA); (2) were aged 18 years or older; (3) had a pre-stroke mRS score ≤ 2. The intracranial internal carotid artery, and M1 and M2 segment of middle cerebral artery were defined as intracranial large vessels. The exclusion criteria were acute and chronic inflammatory diseases such as pneumonia, urinary tract infection, pelvic inflammatory disease and nephritis, autoimmune disease, malignant tumor, or severe renal and hepatic insufficiency. To maintain the homogeneity of the enrolled patients, we excluded patients treated with intra-arterial thrombolysis alone or those diagnosed with concomitant aneurysm or arteriovenous malformation. This study was approved by the ethics committee of each participating center, and due to its retrospective nature; patient consent was waived.

### Baseline Data Collection

Demographics characteristics, clinical data, procedural characteristics and imaging data were recorded after admission. Neurological deficit was assessed by the National Institutes of Health Stroke Scale (NIHSS) (16). The Alberta stroke program early computed tomography Score (ASPECTS) was used to evaluate the extent of preoperative early cerebral ischemia (17). The symptomatic intracranial hemorrhage (sICH) was defined using the criteria of the Heidelberg Bleeding Classification within 24 h after EVT (18). Collateral circulation was determined based on the DSA using the American Society of Interventional and Therapeutic Neuroradiology/Society of Interventional Radiology grading system, with grade 0–1 representing a poor collateral status, and grade 2–4 representing a moderate to excellent status (19). Successful reperfusion was defined as a modified Thrombolysis in Cerebral Infarction score of 2b or 3 (20). Blood samples were obtained from each subject after admission. Laboratory data were also recorded including neutrophil count, platelet count, hyper-sensitive C-reactive protein (hs-CRP), and the lipid profile.

The etiology of target large vessel occlusion was assessed by stepwise angiographic analysis with the results of DSA. If no stenosis was observed and the lumen was smooth, the occlusion was considered to be caused by embolism. ICAS-related occlusion was defined as a remaining stenosis >70%, or a lesser degree of stenosis with a tendency toward re-occlusion or flow impairment during the procedure (7). All neuroimaging data were reviewed by two physicians who were blinded to the clinical data. In case of disagreement, a joint reading was performed, and a consensus decision was reached.

Functional outcome was assessed using the modified Rankin Scale score (mRS) at 90 days after stroke onset. A poor outcome was defined as a modified Rankin Scale score of 3–6. Functional outcome was primarily evaluated by stroke neurologists during the patient's routine clinic follow-up at 3 months. If a patient could not come to the clinic, mRS was determined *via* telephone by interviewing the patient or their family.

### Endovascular Procedures

EVT were performed under local anesthesia or general anesthesia. The type of EVT procedure was selected at the discretion of the treating physician. EVT was performed using different approved modalities, including aspiration with separators, stent retrievers, and aspiration thrombectomy. If recanalization of the targeting artery failed, rescue therapies, such as balloon angioplasty, stent implantation, intra-arterial thrombolysis, or intracatheter tirofiban administration were implemented as needed.

### Statistical Analysis

Continuous variables were reported as means (standard deviation, SD) or medians (interquartile range, IQR). Proportions were calculated for categorical variables. Differences in baseline characteristics between groups were explored using independent sample *t*-tests, Mann–Whitney *U*-tests, chi-square test, or fisher's exact test, where appropriate. To identify the independent risk factors of a poor outcome, a logistic regression model was performed using a forward stepwise method that included all variables with a probability value <0.10 in the univariate analysis. Adjusted odd ratios (OR) and their 95% confidence intervals (CI) were calculated.

To determine whether ICAS could mediate the effect of increased neutrophil count on the risk of poor outcome after EVT, we conducted a mediation analysis to calculate the proportion mediated and to test its significance using the Sobel test (21, 22). A mediation analysis consists of a four-step procedure and seeks to explain a relationship between an independent variable (neutrophil count) and a dependent variable (poor outcome) *via* the inclusion of a third hypothetical variable (presence of ICAS). A two-sided *P* < 0.05 was considered statistically significant. All statistical analyses were performed using SPSS software, version 24.0 (IBM, New York, NY).

## Results

### Patient Baseline Characteristics

**Figure 1:**
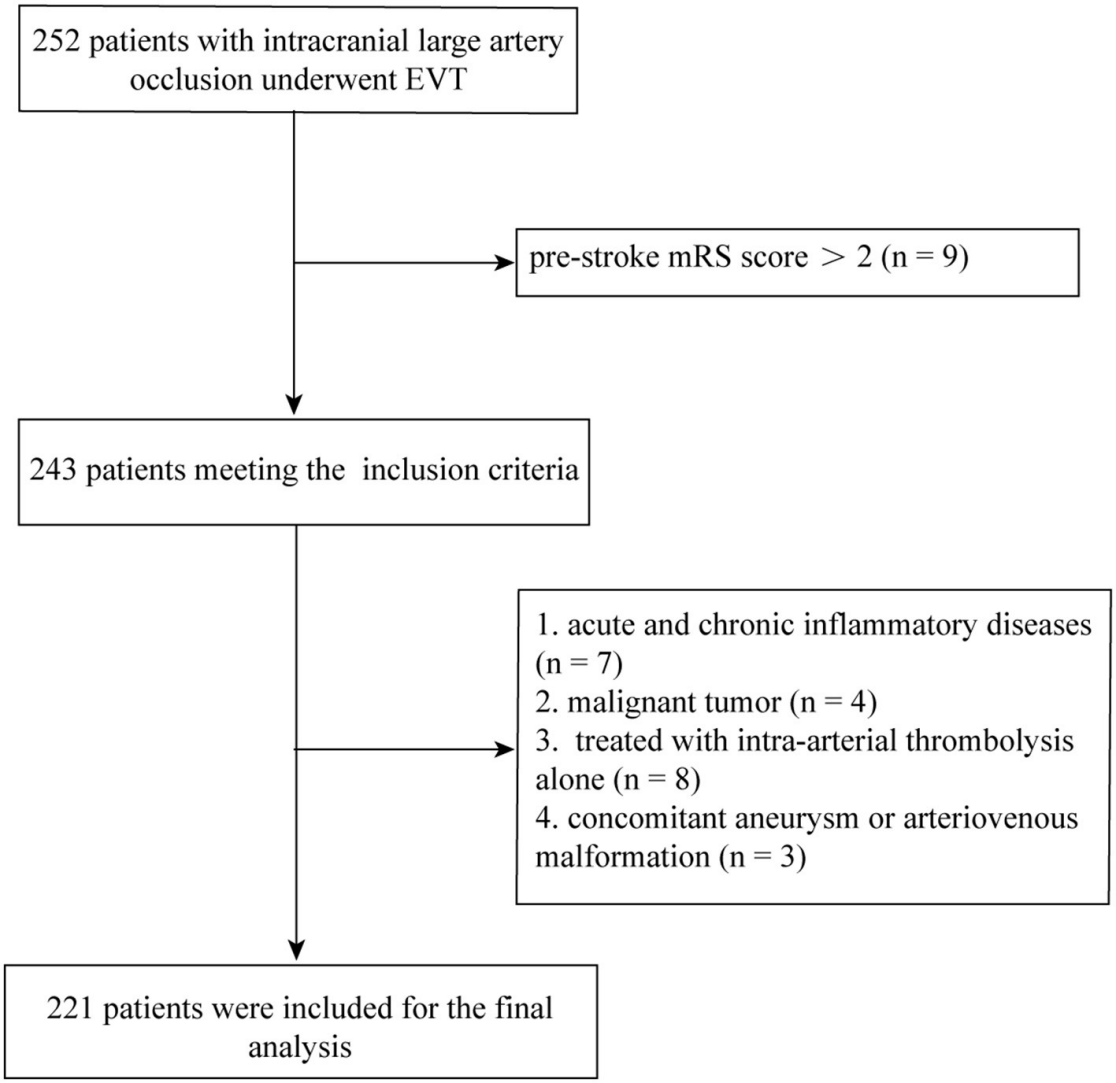
Flow chart of patient inclusion.

**Figure 2:**
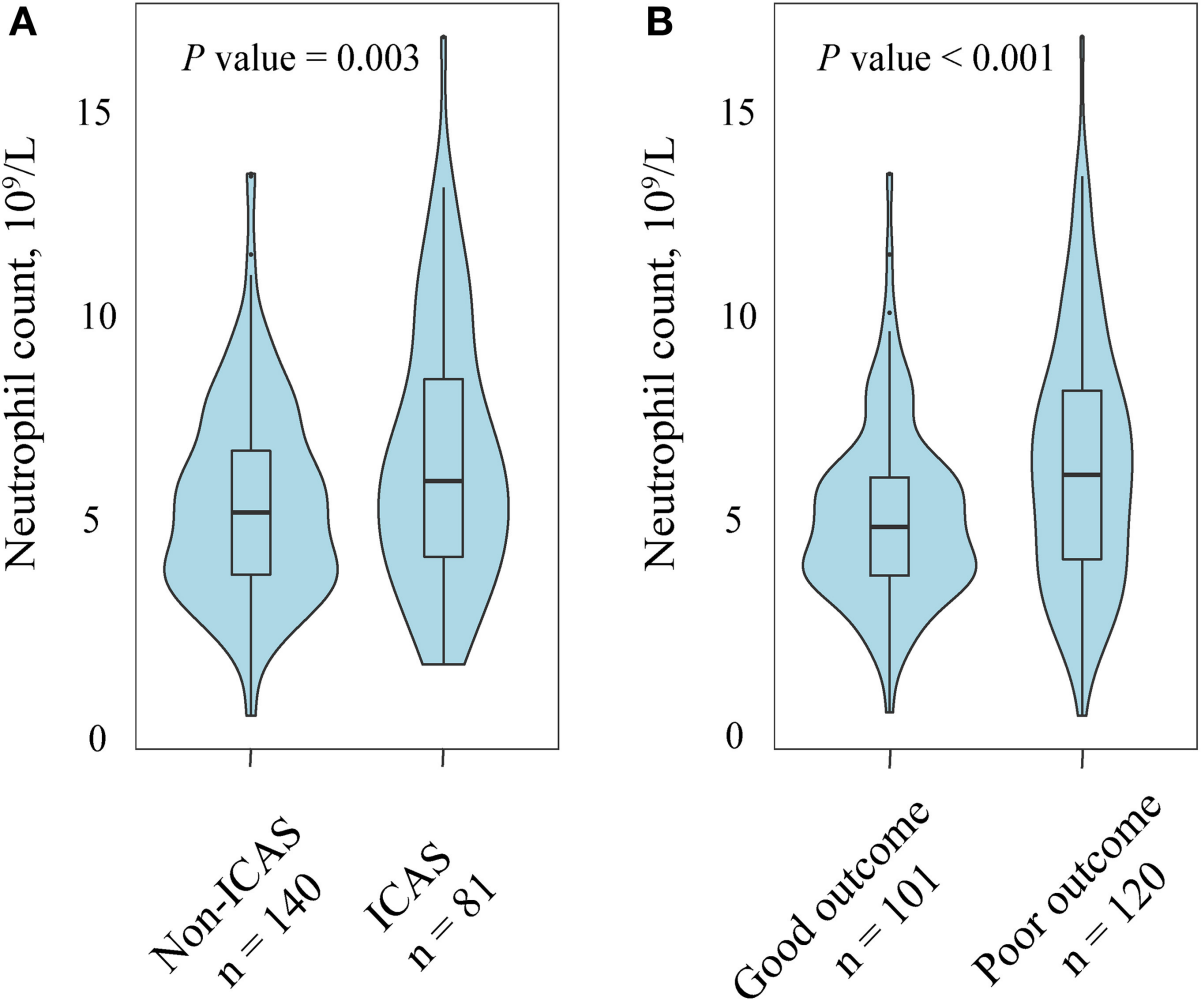
Association of neutrophil count with intracranial atherosclerotic stenosis (ICAS) and functional outcome at 90 days. **(A)** Patients with ICAS tended to have a higher neutrophil count compared with those without ICAS. **(B)** Compared with patients with a favorable outcome patients, the group of patients with an unfavorable outcome had a high level of neutrophil.

A total of 221 patients (mean age, 65.9 years; males, 61.1%) treated with EVT were recruited for the final analysis. The flow chart of patient inclusion is presented in Figure 1. Among them, 81 had ICAS-related occlusion whereas the other 140 had no ICAS (122 for cardioembolic, and 18 for undetermined or others, respectively). General and clinical characteristics of the subjects by median of neutrophil count (5.8 × 10^9^/L) are presented in Table 1. Participants with a higher neutrophil count had an increased baseline NIHSS score (median, 17.0 vs. 14.0; *P* = 0.003), and were more likely to have diabetes mellitus (31.2 vs. 19.6%; *P* = 0.048), ICAS-related occlusion (45.0 vs. 28.6%; *P* = 0.012), and a poor outcome at 90 days (66.1 vs. 42.9%; *P* = 0.001). Similar significant findings were observed when the neutrophil count was analyzed as a continuous variable (Figure 2).

**Table 1:** Comparison of baseline data stratified by the neutrophil count.

**Variables**	**All subjects, *n* = 221**	**Higher neutrophil, *n* = 109**	**Lower neutrophil, *n* = 112**	***P-*value**
**Demographic characteristics**				
Age, years	65.9 ± 13.1	65.8 ± 13.6	66.0 ± 12.7	0.925
Male, *n* (%)	135 (61.1)	67 (61.5)	68 (60.7)	0.909
**Risk factors**				
Hypertension, *n* (%)	137 (62.0)	67 (61.5)	70 (62.5)	0.874
Diabetes mellitus, *n* (%)	56 (25.3)	34 (31.2)	22 (19.6)	0.048
Hyperlipidemia, *n* (%)	20 (9.0)	12 (11.0)	8 (7.1)	0.317
Coronary heart disease, *n* (%)	23 (10.4)	14 (12.8)	9 (8.0)	0.242
Current smoker, *n* (%)	79 (35.7)	36 (33.0)	43 (38.4)	0.405
**Clinical data**				
Systolic blood pressure, mmHg	152.8 ± 23.7	151.8 ± 22.7	153.7 ± 22.8	0.548
Diastolic blood pressure, mmHg	80.5 ± 13.3	79.8 ± 12.6	81.2 ± 14.0	0.448
Onset to groin puncture, min	225.0 (189.0, 270.0)	221.0 (188.0, 283.0)	225.0 (188.5, 250.0)	0.578
Baseline NIHSS, score	15.0 (11.0, 20.0)	17.0 (12.0, 20.0)	14.0 (10.0, 17.0)	0.003
Baseline ASPECTS, score	9.0 (9.0, 10.0)	9.0 (9.0, 10.0)	9.0 (9.0, 10.0)	0.907
ICSA (vs. Non-ICAS), *n* (%)	81 (36.3)	49 (45.0)	32 (28.6)	0.012
ICA (vs. MCA), *n* (%)	84 (38.0)	63 (57.8)	74 (66.1)	0.205
Prior IVT, *n* (%)	148 (67.0)	71 (65.1)	77 (68.8)	0.568
Poor collateral status, *n* (%)	90 (40.7)	40 (40.4)	46 (41.1)	0.915
Stent retriever with rescue therapy, *n* (%)	61 (27.6)	32 (29.4)	29 (25.9)	0.565
Passes with retriever	2.0 (1.0, 2.0)	2.0 (1.0, 2.0)	2.0 (1.0, 2.0)	0.218
Successful reperfusion, *n* (%)	165 (74.7)	76 (69.7)	89 (79.5)	0.106
sICH, *n* (%)	22 (10.0)	15 (13.8)	7 (6.3)	0.062
Poor outcome at 90 days	120 (54.3)	72 (66.1)	48 (42.9)	0.001
**Laboratory data**				
Total cholesterol, mmol/L	4.1 ± 1.0	4.0 ± 0.9	4.1 ± 1.1	0.531
Triglyceride, mmol/L	1.6 (1.3, 3.1)	1.7 (1.3, 2.2)	1.6 (1.3, 1.9)	0.520
Low density lipoprotein, mmol/L	3.0 (2.4, 3.5)	3.0 (2.5, 3.7)	2.9 (2.5, 3.3)	0.235
High density lipoprotein, mmol/L	1.2 (1.1, 1.3)	1.2 (1.1, 1.3)	1.2 (1.1, 1.4)	0.163
Baseline blood glucose, mmol/L	7.9 ± 3.1	8.2 ± 3.1	7.9 ± 3.0	0.504
Hs-CRP, mg/L	1.8 (1.2, 3.5)	1.8 (1.2, 3.9)	2.0 (1.4, 3.2)	0.318
Platelet, 10^9^/L	180.6 ± 70.2	184.3 ± 77.3	176.5 ± 62.7	0.383

*ASPECTS, alberta stroke program early computed tomography score; Hs-CRP, hyper-sensitive C-reactive protein; ICAS, intracranial atherosclerotic stenosis; ICA, internal carotid artery; IVT, intravenous thrombolysis; MCA, middle cerebral artery; NIHSS, national institute of health stroke scale; sICH, symptomatic intracranial hemorrhage*.

### Multivariate Analysis for the Predictors of Poor Outcome at 90 Days

During the 90-day follow-up, 120 (54.3%) patients had a poor functional outcome. A comparison of baseline data in patients with and without an unfavorable outcome at 90 days is shown in Table 2. The univariate analysis revealed that patients with a poor outcome had a higher neutrophil count (mean, 6.7 × 10^9^/L vs. 5.3 × 10^9^/L; *P* < 0.003), onset to groin puncture time (median, 240.0 min vs. 215.0 min; *P* < 0.003), baseline NIHSS score (median, 16.0 vs. 14.0; *P* = 0.002), and passes with retriever (median, 2.0 vs. 2.0; *P* = 0.013); and had a lower ASPECTS (median, 9.0 vs. 10.0; *P* = 0.003) than patients with a favorable outcome. The prevalence of diabetes mellitus (30.8% vs. 18.8%; *P* = 0.041), ICAS-related occlusion (45.0 vs. 26.7%; *P* = 0.005), occlusion site in internal carotid artery (45.0 vs. 29.7%; *P* = 0.022), poor collateral circulation (55.0 vs. 23.8%; *P* < 0.001), sICH (17.5 vs. 1.0%; *P* < 0.001) and unsuccessful reperfusion (41.7 vs. 5.9%; *P* < 0.012) was also significantly higher in patients with a poor outcome than in patients with a favorable outcome.

**Table 2:** Comparison of baseline data in patients with and without unfavorable outcome at 90 days.

**Variables**	**Poor outcome, *n* = 120**	**Good outcome, *n* = 101**	***P-*value**
**Demographic characteristics**			
Age, years	65.8 ± 14.2	65.9 ± 11.8	0.978
Male, *n* (%)	70 (58.3)	65(64.4)	0.360
**Risk factors**			
Hypertension, *n* (%)	78 (65.0)	59 (58.4)	0.315
Diabetes mellitus, *n* (%)	37 (30.8)	19 (18.8)	0.041
Hyperlipidemia, *n* (%)	12 (10.2)	8 (7.9)	0.591
Coronary heart disease, *n* (%)	14 (11.7)	9 (8.9)	0.504
Current smoker, *n* (%)	43 (35.8)	36 (35.6)	0.977
**Clinical data**			
Systolic blood pressure, mmHg	154.6 ± 23.9	150.5 ± 23.4	0.199
Diastolic blood pressure, mmHg	81.4 ± 13.5	79.4 ± 13.1	0.269
Onset to groin puncture, min	240.0 (209.0, 283.0)	215.0 (176.0, 248.0)	<0.001
Baseline NIHSS, score	16.0 (11.0, 21.0)	14.0 (10.0, 17.0)	0.002
Baseline ASPECTS, score	9.0 (8.0, 10.0)	10.0 (9.0, 10.0)	0.003
ICSA (vs. Non-ICAS), *n* (%)	54 (45.0)	27 (26.7)	0.005
ICA (vs. MCA), *n* (%)	54 (45.0)	30 (29.7)	0.022
Prior IVT, *n* (%)	76 (63.3)	72 (71.3)	0.210
Poor collateral status, *n* (%)	66 (55.0)	24 (23.8)	<0.001
Stent retriever with rescue therapy, *n* (%)	35 (29.2)	26 (25.7)	0.571
sICH, *n* (%)	21 (17.5)	1 (1.0)	<0.001
Passes with retriever	2.0 (1.0, 2.0)	2.0 (1.0, 2.0)	0.013
Successful reperfusion, *n* (%)	70 (58.3)	95 (94.1)	<0.001
**Laboratory data**			
Total cholesterol, mmol/L	4.0 ± 1.1	4.1 ± 1.0	0.367
Triglyceride, mmol/L	1.7 (1.2, 2.2)	1.6 (1.4, 1.9)	0.381
Low density lipoprotein, mmol/L	2.8 (2.5, 3.7)	3.2 (2.6, 3.3)	0.671
High density lipoprotein, mmol/L	1.2 (1.1, 1.2)	1.2 (1.1, 1.4)	0.107
Baseline blood glucose, mmol/L	8.3 ± 3.1	7.8 ± 3.2	0.350
Hs-CRP, mg/L	1.8 (1.2, 5.4)	2.3 (1.3, 3.0)	0.892
Platelet, 10^9^/L	179.1 ± 75.4	182.3 ± 63.8	0.743
Neutrophil count, 10^9^/L	6.7 ± 3.0	5.3 ± 2.1	<0.001

*ASPECTS, alberta stroke program early computed tomography score; Hs-CRP, hyper-sensitive C-reactive protein; ICAS, intracranial atherosclerotic stenosis; ICA, internal carotid artery; IVT, intravenous thrombolysis; MCA, middle cerebral artery; NIHSS, national institute of health stroke scale; sICH, symptomatic intracranial hemorrhage*.

**Figure 3:**
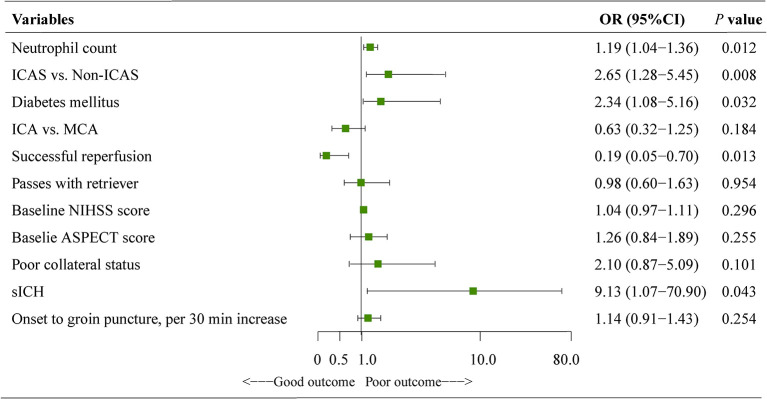
Results of the logistic regression model evaluating the risk factors of poor outcome at 90 days. Multivariate logistic regression was adjusted for potential confounders with *P* < 0.1 in the univariate analysis. ASPECTS, the albert stroke program early CT score; CI, confidence interval; NIHSS, national institutes of health stroke scale; ICAS, intracranial atherosclerotic stenosis; ICA, internal carotid artery; MCA, middle cerebral artery; sICH, symptomatic intracranial hemorrhage.

In the multivariate adjustment for potential confounders (including variables with *P* value < 0.1 in the univariate analysis), neutrophil count (OR, 1.19; 95%CI, 1.04–1.36; *P* = 0.012), ICAS-related occlusion (OR, 2.65; 95CI%, 1.28–5.45; *P* = 0.008), diabetes mellitus (OR, 2.34; 95CI%, 1.08–5.16; *P* = 0.032), successful reperfusion (OR, 0.19; 95CI%, 0.05–0.70; *P* = 0.013), and sICH (OR, 9.13; 95CI%, 1.07–70.90; *P* = 0.043) were significant predictors of a poor outcome (Figure 3).

### Mediation by ICAS-Related Occlusion of the Association Between Neutrophil Count and Functional Outcome

**Figure 4:**
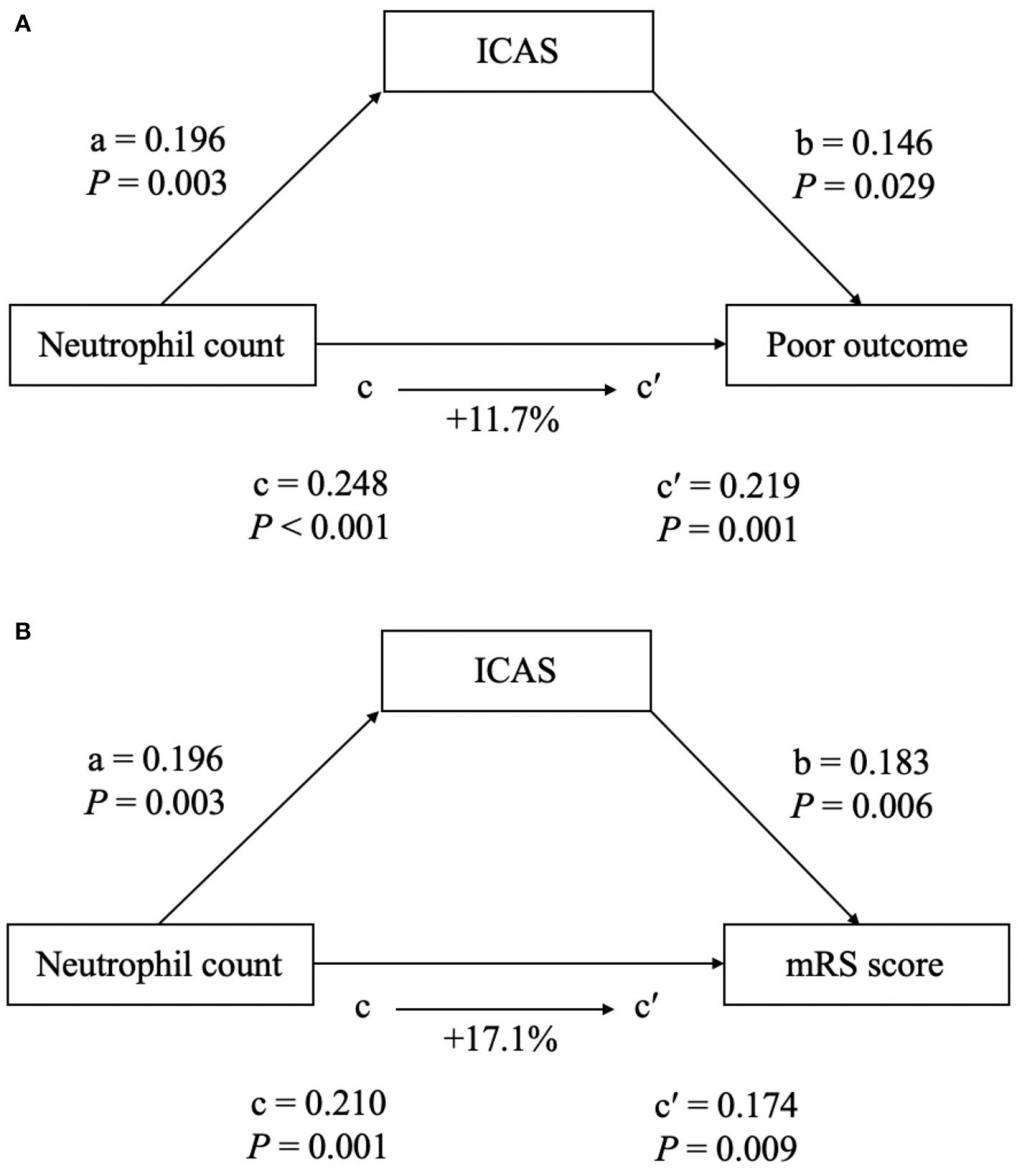
The mediation analysis by intracranial atherosclerotic stenosis (ICAS) of the association between neutrophil count and clinical outcome [**(A)** for poor outcome at 90 days; **(B)** for mRS score at 90 days). a, regression coefficient of the association between neutrophil count and ICAS; b, regression coefficient of the association between ICAS and clinical outcome, using neutrophil count and ICAS as independent variables; c, regression coefficient of the association between neutrophil count and clinical outcome; c′, regression coefficient of the association between neutrophil count and clinical outcome, using neutrophil count and ICAS as independent variables. The percentage difference of the coefficients (1–c/c′) is shown.

To further test the potential mediating effects of ICAS-related occlusion on the association between increased neutrophil count and a worse functional outcome, we conducted a causal mediation analysis. After including ICAS-related occlusion as a mediator, we observed a significant partial mediation effect for ICAS on higher neutrophil count-related effects on the 90-day functional outcome after EVT in ischemic stroke patients. The estimated proportion mediated by ICAS-related occlusion was 11.7% for a poor outcome and 17.1% for the mRS score, respectively (Figure 4).

## Discussion

The results of this observational study demonstrated that an elevated neutrophil count and the presence of ICAS-related occlusion were associated with a higher risk of a 90-day poor outcome in ischemic stroke patients who underwent EVT. Our data further indicated that worse functional outcomes associated with the higher neutrophil count, could be partially explained by the presence of ICAS-related occlusion.

To date only a few studies have reported the clinical outcomes of patients who underwent EVT for ICAS-related occlusion. Furthermore, the results of functional outcomes have been inconsistent (7, 23–25). Data from the Acute Stroke due to Intracranial Atherosclerotic occlusion and Neurointervention-Korean Retrospective registry, found that ICAS-related occlusion could predict the 3-month functional dependence after EVT treatment (7). Also, Lee et al. (25) confirmed that patients with ICAS-related occlusion had much worse outcome than those without it. However, other studies showed no significant difference or a protective factor of ICAS-related occlusion on the prognosis of EVT patients (23, 24). Because the optimal EVT strategy for ICAS-related occlusion was not uniformly established, patients with ICAS-related occlusion were treated according to the neuro-interventionist's judgement, which resulted in a comparable rate of recanalization and poor prognosis. Similar to previous studies that focused on Asian patients, our retrospective multicenter cohort demonstrated that ICAS-related occlusion was associated with an increased risk of a 90-day poor outcome in ischemic stroke patients treated with EVT. The lower rate of recanalization, longer procedure time, and higher rate of using rescue therapy and re-occlusion may partly contribute to the relatively poor outcome in the ICAS-related occlusion after EVT (26).

Mounting evidence has confirmed that inflammation plays a vital role in the worsening of a stroke outcome (27). Our study added to the existing evidence by demonstrating that a higher neutrophil count was positively correlated with an increased risk of a poor outcome among EVT patients. However, the underlying mechanisms by which neutrophils contribute to a worse outcome in patients treated with EVT, remain poorly understood. Neutrophil, as the important factor of inflammatory response, recruits into the ischemic zone and initiates the inflammatory and immune cell migration by releasing detrimental molecules, such as proinflammatory cytokines, chemokines, and reactive oxygen species, which are all involved in infarct growth and are caused symptom deterioration (10, 28). Moreover, in the setting of inflammation, an activated neutrophil can contribute to thrombus formation through neutrophil extracellular traps (NETs) (29). Recent experimental studies reported that ischemic stroke thrombi contain NETs and that their number was associated with the resistance of EVT treatment (12). This could partly explain why an increased neutrophil count is associated with a poor clinical outcome after EVT. In addition, increased neutrophils may contribute to the development and progression of atherosclerosis, and lead to the carotid plaque destabilization (30, 31). Moreover, compelling evidence in total white blood cell count, neutrophil count, and the presence of ICAS have been reported in prior clinical studies (14, 15, 25). Even in a healthy population, the baseline neutrophil-lymphocyte ratio has been reported to be associated with the development of ICAS (15). Therefore, ICAS-related occlusion may play a crucial role in the presence of a poor outcome in EVT patients caused by a higher neutrophil count. Interestingly, our mediation analysis showed that ICAS mediated a modest but significant proportion (11.7% for poor outcome and 17.1% for increased mRS score) of the above-mentioned association, suggesting that atherosclerosis plays an intermediating role. The data from a previous study support the need for new therapies focusing on DMT by targeting NETs, for example, with recombinant DNAse 1 or platelet activation (13). The management of neutrophil counts within an appropriate range could improve the functional outcome after EVT and is a possible future area of inquiry.

Some limitations of the present study must be addressed when interpreting the results. First, this was a retrospective study with data taken from two comprehensive stroke centers, which may have generated system biases. This cohort also included relatively few cases with ICAS-related occlusion. We therefore could not rule out a type I error in the present study. Second, this study did not include all outcome-related variables for mediation analysis, such as blood pressure variation, use of an antiplatelet regime, and infarct volume. Thus, selection biases might have occurred. Third, the neutrophil count was measured only once, at baseline, so we were unable to explore the association between the dynamic change of albumin and prognosis after EVT. Finally, the results of the present study are confined to Chinese patients, and thus, the findings may not be generalized to other ethnicities.

In conclusion, our study found a significant correlation between increased neutrophil count and a higher risk of a 90-day poor outcome after EVT treatment in ischemic stroke patients. The association could be partially mediated by the presence of ICAS-related occlusion.

## Data Availability Statement

The raw data supporting the conclusions of this article will be made available by the authors, without undue reservation.

## Ethics Statement

This study was approved by the Ethics Committee of Shuguang Hospital and Mianyang Central Hospital, and due to its retrospective nature; patient consent was waived.

## Author Contributions

TL, DL, and XY contributed to overall study design. XZ, XT, FG, MW, HZ, YG, JZ, YL, BQ, ZZ, and ZF contributed to clinical data collection. SP contributed to Image data collection. ZC, TL, and DL designed and conduced data analysis. ZC and DL contributed to data analysis and interpretation. ZC wrote the manuscript. WP was involved in the review of the paper. All authors approved the submitted version of the article.

## Conflict of Interest

The authors declare that the research was conducted in the absence of any commercial or financial relationships that could be construed as a potential conflict of interest.
